# Effectiveness of Subconjunctival 0.5% Bupivacaine for Postoperative Analgesia after Intravitreal Silicon Oil Removal Surgery

**DOI:** 10.1155/2018/8501519

**Published:** 2018-12-24

**Authors:** Aida Rosita Tantri, Riyadh Firdaus, Elvioza, Immaculata Astrid Budiman

**Affiliations:** ^1^Department of Anesthesiology and Intensive Care, Faculty of Medicine, Universitas Indonesia, Jakarta, Indonesia; ^2^Department of Ophthalmology, Faculty of Medicine, Universitas Indonesia, Jakarta, Indonesia

## Abstract

**Background:**

Intravitreal silicon oil removal surgery can cause mild-to-moderate postoperative pain and discomfort in most patients. Postoperative pain can be managed by using many methods, including a local anesthetic drug. One of the common local anesthetic drugs is 0.5% bupivacaine. The application techniques also vary, such as subconjunctival application. It was a good alternative for postoperative analgesia in the ophthalmic surgery because of its minimal risks and complications. The purpose of this research was to measure the effectiveness of subconjunctival 0.5% bupivacaine for postoperative analgesia in silicon oil removal surgery.

**Method:**

This was a double-blind randomized clinical study in patients undergoing elective intravitreal silicon oil removal surgery at Cipto Mangunkusumo Hospital. Thirty consecutive patients, enrolled from October 2016 to February 2017, were randomized to receive subconjunctival 0.5% bupivacaine or subconjunctival placebo (0.9% NaCl) at the end of the surgery. The primary outcome was the pain score 24 hours after surgery, using a 100 mm Visual Analogue Scale (VAS). Intravenous injection of tramadol 50 mg was given if the VAS >4. Secondary outcomes were the time to first analgesic requirement and the incidence of nausea/vomiting. Statistical analysis was conducted to measure the difference between 24 h pain score in the bupivacaine group (B) and that in the placebo group (NS).

**Result:**

The overall 24 hours' postoperative pain score was significantly different between the bupivacaine group and the placebo group (*p*=0.001). In the 24 hours after surgery, there were only five patients needing additional analgesia in the placebo group. The time to first analgesic requirement was significantly different between the two groups (*p*=0.042). Nausea/vomiting only happened in the placebo group with proportions 6% and 3%, respectively.

**Conclusion:**

Subconjunctival 0.5% bupivacaine was effective for postoperative analgesia in intravitreal silicon oil removal surgery.

## 1. Introduction

Prevalence of retinal diseases in Indonesia is 8.5% [1, 2]. Although most retinal diseases can be treated without surgery, cases like retinal detachment still need to be surgically repaired.

Retinal surgery takes quite a long time because it includes some procedures and most of the time needs follow-up surgery, intravitreal silicone oil removal, in 3–6 months after the first surgery [3]. Intravitreal silicone oil removal surgery often causes postoperative pain. This is mostly caused by the incision of the periscleral conjunctiva, where the scarring process has already occurred around the incision site from the previous surgery. Besides that, the conjunctiva has a large number of afferent nerve endings from the ophthalmic branch of the trigeminal nerve [4, 5].

Postoperative pain due to intravitreal silicone oil removal surgery is considered mild to moderate pain. The pain would be expressed using a Visual Analogue Scale (VAS) score that ranges from 4 to 6 with the highest intensity occurring in the first 5 hours and decreasing after few hours [6, 7]. Untreated pain will cause several complications such as extended length of stay after surgery, disturbance of wound healing process, surgical wound dehiscence, and transition of acute to chronic pain. Pain management for medical procedures should be done properly to give comfort and satisfaction to patients [8, 9]. Postoperative pain after intravitreal silicone oil removal surgery is generally treated by administration of analgesics such as nonsteroidal anti-inflammatory drugs (NSAIDs), acetaminophen (paracetamol), and weak opioids [6, 10]. According to a preliminary study done in the Operating Room of Kirana, Cipto Mangunkusumo Hospital, in January 2016, 66% of the patients who underwent intravitreal silicone oil removal still complained about the pain with a VAS score that ranges from 2 to 4 and still needed additional analgesics despite previous administration of NSAIDs or paracetamol.

Multimodal analgesia for treating this postoperative pain is very important. One of the techniques is by using local anesthetic drugs as analgesics, as a single agent, or in combination with general anesthesia. Bupivacaine is one of the local anesthetic drugs that belongs to the amino-amides group. It is preferred because it has a quick onset (4–6 minutes) and long duration (120–480 minutes), so it is suitable to treat postoperative acute pain [11].

The application techniques of local anesthetics in eye surgeries vary, such as topical, subconjunctival, peribulbar block, retrobulbar block, and sub-Tenon block [12]. The use of local anesthetic with the peripheral block technique requires skilled operators. Despite its superiority in treating postoperative pain, the peripheral block technique has harmful complications including ocular perforation, bleeding, and nerve optic damage [12, 13]. Therefore, subconjunctival administration of the local anesthetic in the incision site becomes one of the alternatives in treating postoperative pain.

There is still not enough evidence about subconjunctival local anesthetics. HT El-Kasaby et al [14] and Habib et al. [15] reported the use of postoperative subconjunctival administration of 0.5% bupivacaine in strabismus surgery could relieve postoperative pain and reduce the need for analgesics significantly. This study aimed to find out about the effectivity of subconjunctival 0.5% bupivacaine administration as an analgesic after intravitreal silicone oil removal surgery.

## 2. Materials and Methods

### 2.1. Design of Study

This was an experimental study. Subjects were selected consecutively from patients undergoing intravitreal silicone oil removal surgery in the Operating Room of Kirana Eye Center, Cipto Mangunkusumo Hospital, from October 2016 to February 2017. Subjects were divided into two groups with administration of subconjunctival 0.5% bupivacaine (B) for the treatment group and 0.9% NaCl (NS) for the control group. The choices of treatment were randomized using sealed envelopes that were opened right before the anesthesia only by nurse anesthetists who were assigned to prepare the drugs. An anesthesiologist then administered general anesthetic drugs. At the end of the surgery, the ophthalmic surgeon administered either 0.5% bupivacaine or 0.9% NaCl. Both the anesthesiologist and the eye surgeon were unaware of the subconjunctival drug that was administered. Pain intensity using the VAS was recorded to measure the success of anesthesia.

### 2.2. Inclusion and Exclusion Criteria

Inclusion criteria used in this study were patients' age 18–60 years with body mass index (BMI) 18–27.5 kg/m^2^, American Anesthesiologist Association Physical Statuses (ASA-PS) I–II, and signed informed consent to participate in the study. Exclusion criteria were preoperative chronic pain with the use of long-term analgesics before surgery, pregnancy, ambulatory surgery, diagnosed or suspected glaucoma or ocular hypertension, cognitive disturbance, inability to communicate, and additional surgery besides the intravitreal silicone oil removal surgery. Dropout criteria were complications (shock, anaphylactic reaction, and seizure), complications caused by the surgery, and intraocular pressure > 22 mmHg.

### 2.3. Research Protocol

The minimal sample of the study was 30 subjects with 15 subjects within each group. After ethical clearance had been obtained from the Ethical Committee of Cipto Mangunkusumo Hospital, subjects whose informed consent had been obtained were randomized into the 0.5% bupivacaine (B) and 0.9% NaCl (NS) groups. Randomization was done using sealed envelopes opened only by nurse anesthetists who prepared the drugs. After that, general anesthesia inductions were done. At the end of the surgery, assigned treatment was given. Subjects were also given a 20 mg/kgBB dose of paracetamol at the end of the surgery. After that, subjects were transferred to the recovery room (RR). Subjects were observed since they arrived in the RR until 24 hours after surgery, at the 0th minute, 30th minute, 60th minute, 2nd hour, 4th hour, 6th hour, 12th hour, and 24th hour. Their pain intensity was recorded using the Visual Analogue Scale (VAS). If there was breakthrough pain (VAS >4), subjects would get an intravenous 50 mg dose of tramadol and it would be recorded in the data. Postoperative nausea and vomiting (PONV) incidence was also recorded within 24 hours after surgery.

### 2.4. Statistical Analysis

Data analysis was performed using Statistical Package for the Social Sciences (SPSS) for Windows version 21. The VAS score was analyzed using the independent* t*-test if data had normal distribution and the Mann-Whitney test if it had nonnormal distribution.

## 3. Results

**Figure 1:**
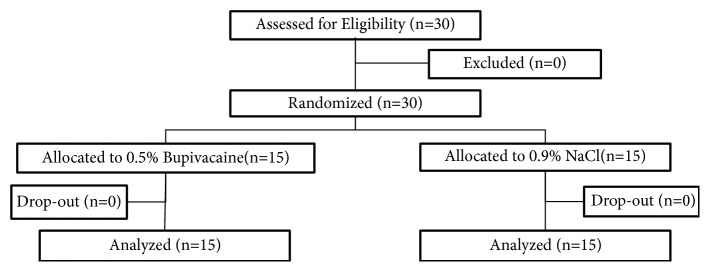
Study flow according to the Consolidated Standards of Reporting Trials (CONSORT) diagram.

There were 30 subjects recruited in this study with 15 subjects in each group. There were no subjects that dropped out during the study as shown in Figure 1.

Characteristics of the study subjects were shown in Table 1. Analysis of baseline data on demographic and clinical characteristics showed both groups were comparable.

Data on pain intensity using the VAS in both groups is shown in Table 2.

The VAS score that was observed 8 times did not have normal distribution, so the data were normalized using log. The normalized data were analyzed using ANOVA for repeated measures and resulted in* p* = 0.001 with estimated power of 99%.

Distribution of the VAS mean from the 0th minute until the 60th minute inclined in both groups. Mean and distribution of the NS group were higher compared to the B group. Distribution of the VAS mean from the 4th hour until the 24th hour in the B group declined, while that of the NS group did not.

In this study, the first time additional analgesics were needed was also recorded.

In the B group, there were no subjects who needed additional analgesics in the 24 hours after surgery. On the other hand, there were 5 subjects who needed additional analgesics in the NS group.

Analysis using Fisher's exact test showed that the number of subjects needing additional analgesics from the two groups was significantly different (*p* = 0.042).

This study also recorded postoperative nausea and vomiting (PONV) incidence in the 24 hours after surgery.

From observation, it was reported that the incidence of PONV was higher in the NS group than the B group.

## 4. Discussion

### 4.1. Characteristics of Study Subjects

Variation of age and gender which were not significantly different will reduce bias in pain perception. It corresponds with the study by Wandner et al. that reported that the women and elderly had a lower pain threshold [16].

ASA-PS in both groups were mostly ASA II, which are patients with controlled hypertension, overweight, and controlled type II diabetes. There were 9 subjects with the shortest surgery duration, which was 15 minutes. That could affect pain intensity after surgery because subjects were still under the effect of the administered intraoperative opioid analgesics. However, the time needed for extubating and transferring patients to the recovery room was estimated to be about 15–20 minutes. The opioid used as an analgesic was fentanyl, which has a half-life of 30 minutes. Therefore, effect of the drug was assumed to have worn off in the recovery room.

### 4.2. Comparison of Pain Intensities Using VAS between 0.5% Bupivacaine and 0.9% NaCl Group

According to a preliminary study done in January 2016, 66% of the subjects undergoing intravitreal silicon oil removal surgery had a VAS score of 2–4 and needed additional analgesics despite of previous administration of NSAIDs or paracetamol. This is in accordance with a previous study by El-Kasaby et al. that reported subconjunctival bupivacaine consistently had better analgesic effect compared to NaCl [14]. Habib et al. also reported postoperative subconjunctival administration of bupivacaine in strabismus surgery could relieve postoperative pain and reduce the need for analgesics significantly [15]. Theoretically it was due to the analgesic effect of bupivacaine which inhibits the pain stimulus caused by the surgical wound. Like other local anesthetic agents, bupivacaine blocks the Na^+^ influx in the Na^+^ channel in the nerve membrane. As a result, it slows down depolarization and inhibits action potential [17, 18].

The VAS score recorded in this study (Table 2) showed more than 2-point difference between the two groups, which were in the 60th minute (2-point difference) and 24th hour (2.2-point difference). The VAS score from the rest of the observation time had less than 2-point difference. However, overall pain intensity and discomfort were significantly reduced in the B group. The variation was due to subjective pain perception from each subject who had a different pain threshold and coping mechanism. Genetics, social, cultural, and psychological factors like previous experience and level of anxiety also played a role in pain perception. The results also could have been different if the type of surgery was more complicated like strabismus or scleral buckling surgery [19, 20].

In the NS group, the VAS score reached the highest peak in the 60th minute. There were 3 subjects whose VAS scores were 7. It may be due to subject transfer from the recovery room to the ward so this affected the pain intensity.

**Figure 2:**
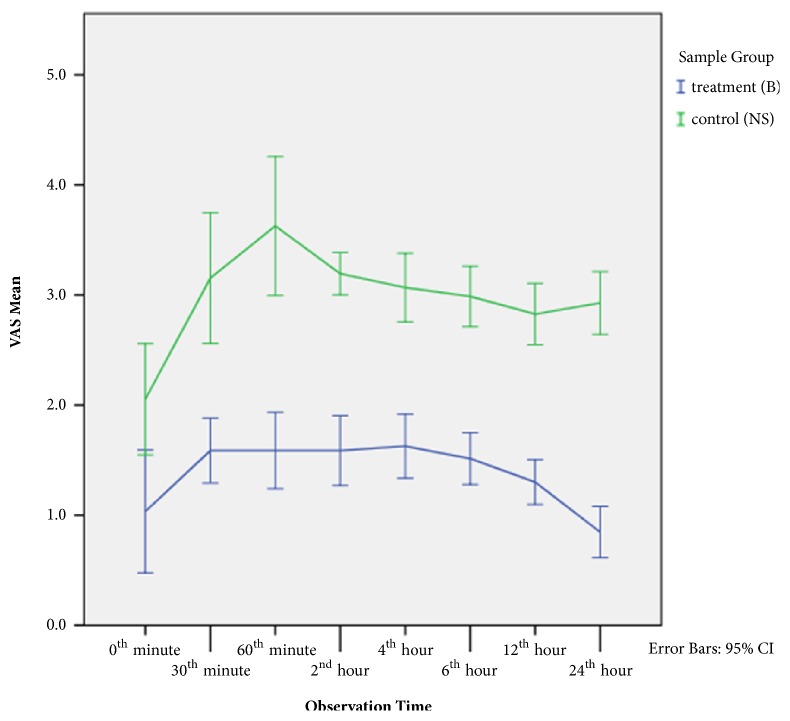
Comparison of mean and distribution of VAS scores 24 hours after surgery between the bupivacaine group (B) and NaCl group (NS).

Overall mean of the VAS score in the NS group was higher than in the B group in the 24 hours after surgery (Figure 2). After the 60th minute, mean of the VAS score in the NS group declined due to administration of additional analgesics, tramadol. Duration of action of tramadol is 6–8 hours, so it was assumed that a high VAS score recorded in the 12th and 24th hour was not affected by previously administered additional analgesics. The high VAS score in the NS group proved that bupivacaine was a potent postoperative drug that had analgesic effects for up to 24 hours after surgery.

The conjunctiva consists of two layers, an outer layer made up of stratified epithelium and an inner layer made up of nerves, blood vessels, and lymph nodes. The adherens junction (a.k.a. zonula adherens) with its transepithelial electrical resistance in this structure plays a role as a barrier from hydrophobic drugs [20]. For this reason, in the B group, mean of the VAS score reached the highest peak in the 30th minute and then started declining in the 4th hour to 24th hour (Figure 2). It was due to administration of bupivacaine that gradually took effect. Duration of action of bupivacaine is 4–8 hours, but the layered structure of the conjunctiva allows bupivacaine to last longer and not be easily diffused to deeper structures [21, 22]. Besides that, the low molecular weight of bupivacaine (0.288 kDa) facilitates its absorption and distribution into the eye structure. The high protein binding capacity of bupivacaine (95%) [18] also inhibits its absorption into the systemic circulation. This explains how subconjunctival drugs are deposited and work longer.

In this study, the number of subjects that needed additional analgesics (Table 4) was significantly higher in the NS group (5 subjects) compared to the B group (0 subjects). The earliest time an additional analgesic was needed (Table 3) was in the 30th minute (1 subject), followed by the 60th minute (3 subjects) and the 4th hour (1 subject).

The incidence of postoperative nausea and vomiting (PONV) was higher in the NS group than the B group (Table 5). This was probably due to administration of additional analgesics (tramadol) when breakthrough pain happened. This is in accordance with a study by Odom-Forren et al. that reported subjects with high pain intensity had a greater nausea degree compared to subjects with low pain intensity [23]. Correlations between pain and PONV were influenced by several factors. According to Miller, brain areas that have roles in regulating nausea and vomiting are the brain stem near the retrofacial nucleus, the postrema area, the solitary tract nucleus, and the dorsal motor nucleus of the vagus nerve. It is predicted that serotonin and NK-1 receptors are involved in the central mechanism of vomiting [24, 25]. Moreover, vomiting is triggered by noxious stimuli that cause release of serotonin which activates the peripheral receptors and visceral afferents that send feedback to the brain stem. Activation of *α*2-adrenergic receptors on the chemoreceptive trigger zone (CTZ) also causes vomiting. Besides that, autonomic imbalance, such as surgical stress response and postoperative pain, is also involved in the mechanism of nausea and vomiting [24, 25].

### 4.3. Limitations of the Study

The limitation in this study lies in the type of eye surgery done. We only studied intravitreal silicon oil removal surgery which has a short duration (<30 minutes). Thus, effectivity of subconjunctival bupivacaine as an analgesic in longer and more complex procedures has yet to be extensively studied. Moreover, effectiveness of the drug was only assessed by using the VAS mean and the earliest time additional analgesics were needed. We did not assess patient satisfaction with the pain management.

## 5. Conclusion

Administration of subconjunctival 0.5% bupivacaine was effective as a postoperative analgesic in intravitreal silicon oil removal surgery. The average of pain intensity in the 24 hours after surgery was significantly different in the group with administration of subconjunctival 0.5% bupivacaine (treatment group) compared to that with 0.9% NaCl (control group). Unlike the control group, the treatment group did not need any additional analgesics. There was also no incidence of PONV found in the treatment group.

## Figures and Tables

**Table 1:** Characteristics of subjects.

**Data**	**B Group (n=15)**	**NS Group (n=15)**
Age (years)	46 ± 10	45 ± 10
Gender		
Male	8 (53.3%)	9 (60%)
Female	7 (46.7%)	6 (40%)
ASA-PS		
I	3 (20%)	4 (26.7%)
II	12 (80%)	11 (73.3%)
Weight (kilogram)	72 ± 15	64 ± 9
Height (centimeter)	163 ± 10	161 ± 7
BMI (kg/m^2^)	26.61 ± 3.23	24.93 ± 3.07
Surgery duration (minute)	20 (15-55)	20 (15-50)

Data are presented in average ± SD and N (%). If distribution was not normal, data are presented in median (min-max).

**Table 2:** VAS score in in the 0th minute, 30th minute, 60th minute, 2nd hour, 4th hour, 6th hour, 12th hour, and 24th hour after surgery.

**VAS**	**B Group**	**NS Group**	**p** ^**c**^
0th minute	1 (0-3.5)	2.2 (1 -3.6)	0.012
30th minute	1.5 (0.8-2.5)	2.7 (2.1-6.5)	<0.001
60th minute	1.4 (0.5-2.7)	3.4 (2.3 -7)	<0.001
2nd hour	1.4 (0.8-2.9)	3.3 (2.4-3.6)	<0.001
4th hour	1.6 (0.8-2.7)	3.1 (2.1-4.4)	<0.001
6th hour	1.5 (1-2.4)	3.0 (2.0-3.8)	<0.001
12th hour	1.2 (0.7-2.0)	3.0 (2.0-3.6)	<0.001
24th hour	0.8 (0-1.7)	3.0 (2.2-3.6)	<0.001

^c^Mann-Whitney test, significant if *p* value < 0.05. Data are presented in median (min-max) because the distribution was not normal.

**Table 3:** First time additional analgesics were needed after surgery.

**Analgesics**	**B Group**	**NS Group**
	**(n=15)**	**(n=15)**
First time additional analgesics were needed (minute)	n/a	30' (n=1)
		60' (n=3)
		240' (n=1)

**Table 4:** Number of subjects who needed additional analgesics.

		**Sample Group**				
		**B Group**		**NS Group**		***p* value**
		**n**	%	**n**	%	
**Needed additional analgesics**	No	15	100.0%	10	66.7%	0.042
	Yes	0	0.0%	5	33.3%	

Analyzed using Fisher's exact test, significant if *p* <0.05.

**Table 5:** Incidence of PONV after surgery.

**Side effects**	**B Group**	**NS Group**
	**(n=15)**	**(n=15)**
Nausea	0 (0%)	3 (20%)
Vomiting	0 (0%)	1 (6.7%)

## Data Availability

The data used to support the findings of this study are available from the corresponding author upon request.
